# The Antibacterial and Remineralizing Effects of Biomaterials Combined with DMAHDM Nanocomposite: A Systematic Review

**DOI:** 10.3390/ma14071688

**Published:** 2021-03-30

**Authors:** Alison Clarin, Daphne Ho, Jana Soong, Cheryl Looi, Deepak Samuel Ipe, Santosh Kumar Tadakamadla

**Affiliations:** 1 School of Dentistry and Oral Health, Griffith University, Gold Coast 4217, Australia; alison.clarin@griffithuni.edu.au (A.C.); daphne.ho@griffithuni.edu.au (D.H.); jana.soong@griffithuni.edu.au (J.S.); cheryl.looi@griffithuni.edu.au (C.L.); deepu313@gmail.com (D.S.I.); 2 Menzies Health Institute Queensland, Gold Coast 4217, Australia

**Keywords:** dental materials, composite resin, antibacterial, remineralization

## Abstract

Researchers have developed novel nanocomposites that incorporate additional biomaterials with dimethylaminohexadecyl methacrylate (DMAHDM) in order to reduce secondary caries. The aim of this review was to summarize the current literature and assess the synergistic antibacterial and remineralizing effects that may contribute to the prevention of secondary caries. An electronic search was undertaken in MEDLINE using PubMed, Embase, Scopus, Web of Science and Cochrane databases. The initial search identified 954 papers. After the removal of duplicates and screening the titles and abstracts, 15 articles were eligible for this review. The amalgamation of 2-methacryloyloxyethyl phosphorylcholine (MPC) and silver nanoparticles (AgNPs) with DMAHDM resulted in increased antibacterial potency. The addition of nanoparticles of amorphous calcium phosphate (NACP) and polyamidoamine dendrimers (PAMAM) resulted in improved remineralization potential. Further clinical studies need to be planned to explore the antibacterial and remineralizing properties of these novel composites for clinical success.

## 1. Introduction

Dental composite resins are widely used restorative materials due to their ability to conserve tooth structure during cavity preparation, aesthetics and direct-filling capabilities [1,2,3,4]. However, composite resin restorations still present several drawbacks including polymerization shrinkage, bulk fracture, mechanical fatigue by mastication, marginal leakage and biodegradation by acid while also being challenged by the adhesion and accumulation of cariogenic bacteria (Viz., *Streptococcus mutans* and *Lactobacilli*) when compared to other materials used for restoration [1,2,5].

Dental biofilm adjoining to the tooth-restoration margin could lead to secondary caries, the primary reason for failure of composite restorations [4], accounting for nearly 50% of failures within 10 years [6,7,8]. The replacement of failed restorations may result in further tooth structure removal, which can weaken the remaining hard tissues and affect the long-term prognosis of the tooth [9]. Consequently, efforts have been made to create a novel dental composite resins with the addition of antibacterial and remineralizing agents to combat secondary caries [1].

When copolymerized in resins, quaternary ammonium methacrylates (QAMs), which are cationic compounds, exhibit low toxicity and a broad-spectrum antimicrobial effect [2,10,11,12]. These positively charged methacrylic monomers bind and disrupt the electrical equilibrium of the negatively charged bacterial membranes thereby causing rupture and cell death [13].

The antibacterial efficacy of QAMs has been correlated with the alkyl chain length (CL) of the hydrocarbons as this causes an increase in hydrophobicity [2,14,15]. Previous studies have reported that an increase in CL from 3 to 16 greatly enhanced the efficacy of dental materials against bacteria [16], which then decreased at a CL of 18 [17,18]. A CL of 18 exhibited an increase in live bacteria and biofilm thickness and did not further decrease the metabolic activity or strengthen the bacterial inhibition effect compared to a CL of 16 [19,20]. Therefore, dimethylaminohexadecyl methacrylate (DMAHDM) monomer with a CL of 16 was found to exhibit the best antiseptic effect against oral bacterial pathogens when used as bonding agents, sealers or other dental materials [17,21].

Due to the strong antibacterial properties of DMAHDM, researchers have experimented with combining additional biomaterials with DMAHDM to explore any synergistic mechanisms of action to increase the efficacy of DMAHDM and reduce biofilm formation at the tooth-restoration interface [8,11]. DMAHDM does not possess any inherent remineralizing capabilities, so efforts have also been made to incorporate biomaterials that promote tooth remineralization as another approach for caries inhibition [1,8,22]. Furthermore, the development of this nanotechnology is quickly evolving and there has been a shift in focus to create novel composite resins that possess both antibacterial and remineralizing capabilities [23].

Despite this growing area of research, there is yet to be a review that outlines the current combinations of DMAHDM nanocomposite or any synergistic mechanisms of action that enhance the effects of DMAHDM nanocomposite. As such, this systematic review intended to summarize the literature on dental composite resins that incorporate additional biomaterials with DMAHDM, and to assess for any possible synergistic antibacterial and remineralizing effects that may aid in the prevention of secondary caries.

## 2. Materials and Methods

Preferred reporting items for systematic review and meta-analyses (PRISMA) guidelines [24] have been followed to report this review. The protocol of this review registered on the international prospective register of systematic reviews (CRD42020198770).

### 2.1. Eligibility Criteria

Eligible studies included those that assessed the antibacterial, remineralization and/or anticaries potential of composite resins, which combined DMAHDM with additional biomaterials. This review was to determine whether the inclusion of bioactive materials could aid in the prevention of secondary caries through antibacterial and/or remineralizing effects. Studies that reported on dental materials other than composite resin, assessed QAMs other than DMAHDM, or assessed properties other than antibacterial/remineralization/anti-caries potential, were excluded. Animal studies and review articles were also excluded. All relevant articles were found to be in-vitro studies and no clinical studies were found.

### 2.2. Search Strategy and Study Selection

An electronic search of the literature was undertaken and included all relevant studies up to April 2020 in MEDLINE using PubMed, Scopus, Web of science, Embase and Cochrane. No date or language limitations were placed during the search. The full electronic search terms used for in PubMed are shown in Table 1. No further search filters were used during the search.

Two reviewers (AC and CL) screened all the retrieved studies’ titles and abstracts against the inclusion and exclusion criteria independently. Those articles found relevant were independently reviewed in full by two reviewers (DH and JS) to confirm the eligibility. Any disparities between the reviewers’ opinions on inclusion were resolved through discussion or by involving other reviewers.

### 2.3. Data Extraction

Relevant data were extracted using pre-established extraction charts by two reviewers (JS and DH) independently. Extracted data were checked for accuracy by two different reviewers (AC and CL). Authors were contacted if there were any missing data or additional data was required from the eligible studies. The following data items were extracted from the eligible studies: author (year); study design and setting; aims or objectives; type of biomaterials used in the test and comparison groups; method of collecting the clinical sample and assessing the outcomes; results related to antibacterial, remineralization and anticaries outcomes and study duration.

### 2.4. Risk of Bias in the Included Studies

The method of assessing the risk of bias was adopted from a previous review [25,26]. Two reviewers (AC and CL) evaluated the risk of bias by assessing the reporting within the studies on the following seven criteria: sample size calculation; adequate control group; use of materials to the manufacturer’s instructions; standardized sample production process; standardized antibacterial assessment and samples production process; evaluation of antibacterial properties by single operator and adequate statistical analysis. For each criterion reported, a score of “1” is provided when the response is “yes” or the criteria is addressed while a score of “0” is given when the response is “no”, i.e., when the criteria is not addressed in the study. The possible scores for each study could range from 0 to 7. Studies that met six or seven of the criteria were categorized as low risk of bias, those meeting 4–5 criteria as medium risk and one to three criteria as high risk. A third reviewer (DH) was involved when there were any differences in assessments among the reviewers.

## 3. Results

### 3.1. Study Selection

**Figure 1:**
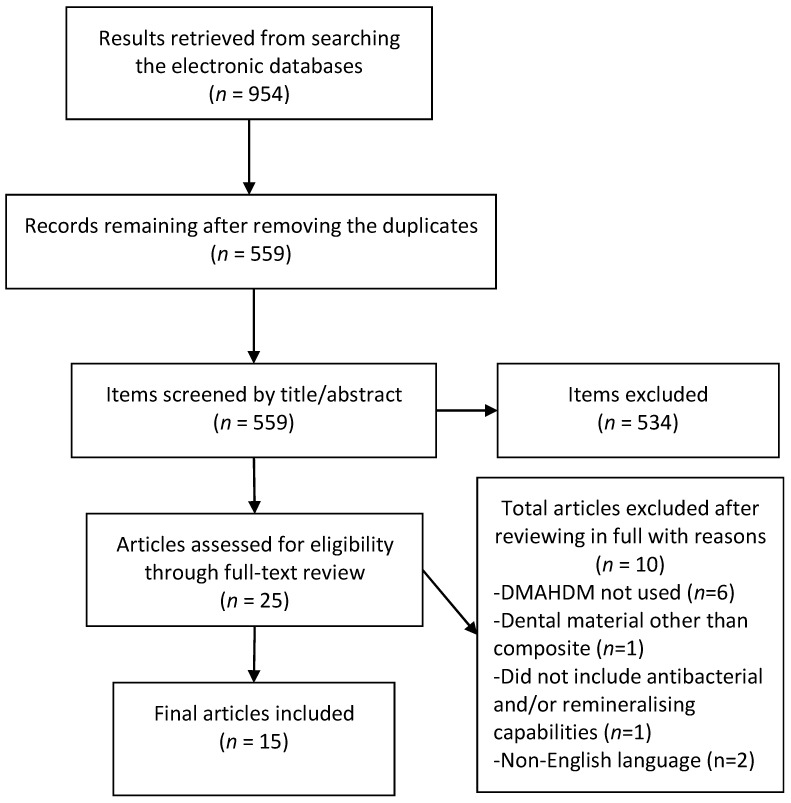
Preferred reporting items for systematic review and meta-analyses (PRISMA) flow chart demonstrating the selection of articles.

The initial literature search retrieved 954 results, 559 remained after removal of duplicates. After screening of titles and abstracts 25 articles remained, of which 15 were eligible for inclusion after the full-text review. Six articles were excluded as they did not use DMAHDM or had an additive biomaterial [27,28,29,30,31,32], one each used dental materials other than the composite [33] or tested materials with compromised mechanical properties [34] while two were non-English articles [35,36]. The results of the screening and search process are presented in Figure 1.

### 3.2. Study Characteristics

All study characteristics are reported in Supplementary Materials Table S1. There were no studies published prior to 2015. All the studies were conducted in USA and/or China, or Japan. Eight of the studies used EBPM (ethoxylated bisphenol A-dimethacrylate (EBPADMA) and pyromellitic glyceroldimethacrylate (PMGDM)) as the composite resin matrix while seven of the studies used a BisGMA-TEGDMA (bisphenol A-glycidyl methacrylate and triethylene glycol dimethacrylate) resin matrix. Nine studies used the heliomolar commercial composite as a control comparison group, two studies used the Renamel Microfill commercial composite and one study used both as a control group. 

There were six different combinations reported in the literature, which incorporated additional biomaterials with DMAHDM: DMAHDM + nanoparticles of amorphous calcium phosphate (NACP); DMAHDM + 2-methacryloyloxyethyl phosphorylcholine (MPC); DMAHDM + NACP + MPC; DMAHDM + NACP + silver nanoparticles (AgNPs); DMAHDM + NACP + MPC + AgNPs and DMAHDM + NACP + MPC + AgNPs + polyamidoamine dendrimers (PAMAM). 

All studies used a modified Menschutkin reaction method to synthesize DMAHDM [2]. NACP was synthesized with a spray-drying technique [37]. MPC was sourced commercially. AgNPs was chemically prepared by dissolving silver 2-ethylhexanoate in 2-(tert-butylamino)ethyl methacrylate (TBAEMA) [38]. PAMAM was used at 1 mg/mL concentration, by mixing PAMAM in distilled water [23]. Antibacterial and remineralizing outcomes used by the included studies are outlined in Table 2.

### 3.3. Risk of Bias

As reported in Table 3, all 15 studies demonstrated a medium risk of bias and reported an adequate control group, standardized sample production process, standardized antibacterial/remineralizing assessment and adequate statistical analysis. No studies reported that the antibacterial or remineralizing properties were evaluated by a single operator or presented a sample size calculation. No studies reported about the manufacturer’s instructions for using the materials. As these are experimental studies, most studies prepared their own composite materials.

### 3.4. Antibacterial Results

There were 14 studies that assessed the antibacterial potential of incorporating bioactive materials with DMAHDM to target specific bacterial species (Table 4). Seven studies assessed efficiency against all bacterial species, all streptococci and *Streptococcus mutans*. One study assessed efficiency against *S. mutans*, *Lactobacillus acidophilus*, *Candida albicans* and multispecies biofilms. Additionally, periodontal pathogens found in subgingival biofilms are highly associated with periodontal disease and root caries. Four studies looked at the antibacterial efficiency against specific root caries pathogens [48], including *Prevotella. intermedia*, *Porphyromonas. gingivalis* and *Aggregatibacter. actinomycetemcomitans*, with an objective of using this restorative material for cervical restorations. One study did not specify the microorganisms tested.

#### 3.4.1. Live/Dead Staining

All 14 articles that evaluated antibacterial potential assessed the biofilms for live/dead staining on the samples of composites. After staining, green and red fluorescence indicated live and dead bacteria respectively; this was then evaluated with an inverted epifluorescence microscope [43]. All studies reported that composites with DMAHDM had significantly reduced live bacteria than control composites. Among the ten studies that assessed the antibacterial potential of NACP alone, all reported similar results to control composites where bacteria were primarily alive, indicating NACP did not have any additional antibacterial effects with DMAHDM [1,2,37,42,46]. Composites with MPC alone showed lower levels of bacterial adhesion but the bacteria were found to be mostly alive; however, when combined with DMAHDM, there were less biofilms and the bacteria had mostly compromised cell membranes [3,38,40,43,44,45] with no significant changes in results after 180 days of water-aging [45].

#### 3.4.2. CFU Counts

There were 13 articles that assessed the CFU counts to understand the antibacterial effect of DMAHDM composite with or without additional bioactive materials. All studies reported a decrease in CFU counts for all microorganisms tested against composites containing DMAHDM compared to control composites or composites without DMAHDM, confirming its antibacterial potency. One study reported increased antibacterial activity (decreasing CFU counts) with an increase in the mass fraction of DMAHDM [39]. Adding MPC to the DMAHDM composite had a synergistic antibacterial effect that decreased CFU counts much more than using either biomaterial alone as observed by three studies [40,44,45]. One study described a synergistic effect against CFU counts for *Fusobacterium nucleatum*, *P. gingivalis* and *A. actinomycetemcomitans* when incorporating 0.12% AgNPs into the composite [38].

#### 3.4.3. MTT Metabolic Assays

Eleven articles assessed metabolic assays where the biofilms were incubated with MTT solution, which resulted in the bacteria that were metabolically active. Four studies reported that the metabolic activity of biofilms on DMAHDM composites was lower than commercial controls or NACP alone, which had little effect on biofilm viability [1,37,39,47]. Five studies showed that DMAHDM + MPC combinations resulted in the least metabolic activity of all biofilms including total microorganisms or total *streptococci*, and all periodontal pathogens [40,43,44,45,46]. One study explained that DMAHDM + MPC was effective in killing single or polymicrobial biofilms, compared to DMAHDM alone whose killing efficacy decreased as the number of species in the biofilm increased [46]. Xiao et al. (2019) reported that the addition of 0.12% AgNPs reduced the metabolic activity of biofilms for periodontal pathogens (*F. nucleatum*, *A. actinomycetemcomitans* and *P. gingivalis*) compared to composites without AgNPs [38].

#### 3.4.4. Lactic Acid Production

Eight articles assessed the production of lactic acid from biofilms by adding buffered-peptone water to the composite disks and incubating them. Lactic acid production was significantly lower in the DMAHDM group than the NACP composite group [1,2,20,37,47]. NACP alone produced higher levels of lactic acid production that were comparable to commercial control composites [20,47]. It was also reported by two studies that DMAHDM + MPC had the least lactic acid production compared to using the biomaterials alone [40,45].

#### 3.4.5. Protein Adsorption

The protein adsorption of biofilms was evaluated by six studies by using a micro bicinchoninic acid (BCA) protein assay. One study reported that DMAHDM + MPC had less protein adsorption compared to that of the biomaterials alone [40]. However, four studies reported that DMAHDM alone had no significant effects on protein adsorption, whereas MPC substantially decreased the protein adsorption [43,44,45,46], with no difference in efficacy over 180 days [45]. A synergistic effect was found when incorporating 0.12% AgNPs, which significantly reduced protein absorption compared to other concentrations of AgNPs as reported by Xiao et al. (2019) [38].

#### 3.4.6. Polysaccharide Production

Polysaccharide production in biofilms’ extracellular polymeric substance (EPS) was assessed by five articles. Composites containing DMAHDM greatly reduced polysaccharide production for all biofilms compared to NACP composites, which had similar polysaccharide production to commercial control composites [38,42,47]. MPC alone also significantly reduced polysaccharide production, however, a combination of DMAHDM + MPC displayed a synergistic effect exhibiting the least amount of polysaccharides for all types of biofilms tested [43,46]. Furthermore, Xiao et al. (2019) also described a synergistic effect by adding 0.12% AgNPs, which had a further reduction of polysaccharides than DMAHDM + MPC [38].

#### 3.4.7. Other Results

One study tested the pH for the media that was incubated with bacterial biofilm and composite disks with or without bioactive materials [44]. Xie et al. (2016) reported that DMAHDM + MPC composite maintained a safe pH of >6.5, in comparison to the cariogenic pH of 4.2 in the control group [44]. Wang, Melo, et al. (2016) evaluated biofilm biomass and reported that DMAHDM had a significantly decreased biofilm biomass value compared to NACP composite and commercial control composite [42].

### 3.5. Remineralization Results

There were five studies that assessed the remineralization potential of incorporating bioactive materials with DMAHDM (Table 5). All five studies incorporated NACP as a remineralizing agent, while one study also incorporated MPC, AgNPs and PAMAM to assess its remineralizing potential. 

#### 3.5.1. Calcium and Phosphate Ion Release

Five studies evaluated and compared the levels of calcium (Ca) and phosphate (P) ion release. The total time before evaluating varied from 21 to 70 days. One early study reported a moderately lower Ca and P ion release in DMAHDM + NACP composite group than the NACP composite group [20]. This was contradicted by three later studies, which found no significant difference between the NACP and DMAHDM + NACP composites [1,2,47]. Xiao et al. (2017) reported that DMAHDM + MPC + NACP + AgNPs and DMAHDM + MPC + NACP + AgNPs + PAMAM had higher calcium and phosphate concentrations than other control groups [23]. Two articles assessed ion recharge and rerelease and Ca and P ions. Both studies reported that NACP and DMAHDM + NACP continuously released ions after being recharged with no significant differences between them [1,2].

#### 3.5.2. Other Results

Two studies evaluated dentine hardness at the dentine-restoration interface. Xiao et al. (2017) concluded that DMAHDM + MPC + NACP + AgNPs + PAMAM had the greatest dentine hardness, remineralization and mineral growth. Zhou et al. (2020) found that dentine hardness in DMAHDM + NACP group was more than double that of the control groups [23]. Xiao et al. (2017) further analyzed acid neutralization and a scanning electron microscopic examination (Table 5). It was reported that DMAHDM + MPC + NACP + AgNPs and DMAHDM + MPC + NACP + AgNPs + PAMAM had greater acid neutralization than PAMAM alone or other control groups [23].

## 4. Discussion

The aim of this systematic review was to report the current combinations of the DMAHDM composite and to assess the synergistic effects on the prevention of secondary caries. The findings from the studies included indicate that incorporating additional biomaterials with DMAHDM produces a positive synergistic effect on the prevention of secondary caries through antibacterial and remineralizing capabilities.

Regardless of the type of biomaterial combination added to the composite, all studies considered in this review reported a strong antibacterial efficacy of DMAHDM alone, with one study observing increased potency as the mass fraction increased [39]. It is suggested that the mechanism of action of QAMs is through contact inhibition leading to cell death [43,44]. However, it is essential that salivary protein adsorption occurs on the composite surface for bacterial adhesion to follow, and this pellicle separating the resin surface from the biofilm could decrease QAM’s antibacterial efficacy [3,23,43].

MPC is a biocompatible polymer that was shown to have a synergistic mechanism of action when incorporated with DMAHDM compared to either agent alone [38,40,43,44,45,46]. Due to its hydrophilic surface, MPC can inhibit bacterial adhesion and decrease protein adsorption [38,43,49], thus, forming direct contact of the resin surface with the overlaying biofilms and enhancing the contact-killing mechanism of DMAHDM [38,43].

The literature reported DMAHDM + MPC having less biofilms with mostly compromised cell membranes, decreased CFU counts, the least metabolic activity, stronger killing efficacy, least lactic acid production, decreased protein adsorption and maintained a pH above 6.5 [44]. However, Zhang et al. (2017) observed live bacteria on the MPC + DMAHDM composite despite a reduction in lactic acid production from biofilm and total microorganism CFU counts [45]. This further emphasizes the need for more research into the long-term effectiveness of these novel composites on the prevention of secondary caries in orally healthy individuals.

Several articles also reported on the efficacy of novel composites against root caries pathogens [23,38,42,43,46,47]. Composites containing DMAHDM and MPC produced significantly lower levels of lactic acid from *S. mutans* and polymicrobial biofilms [1,2,20,37,47]. Lactic acid is a known byproduct of caries-causing bacteria, and coupled with their aciduric properties, allows them to survive in low pH conditions [47]. By reducing the amount of lactic acid, root dentine demineralization at the restoration margins may be reduced [47], which can improve the longevity of these restorations.

Combining MPC with DMAHDM was effective in inhibiting extracellular matrix synthesis of root caries pathogens by having substantially reduced polysaccharide production [47]. Polysaccharides are a key component of the extracellular polymeric substances (EPS) that surrounds biofilms and protects pathogens from antibacterial agents. Reducing polysaccharide production reduces this protection and the virulence of these pathogens, potentially reducing caries and inhibiting local periodontitis [50].

AgNPs have been associated with many dental applications such as acrylic resins for dentures [51], endodontic irrigants and intracanal medications [52,53] and other restorative materials [15,54]. The antibacterial properties of AgNPs is hypothesized to be due to the release of silver ions to the bacterial environment where the nanoparticle promotes infiltration into the bacterial cell membranes and affects intracellular processes [41]. Furthermore, the incorporation of AgNPs at 0.12% concentration with MPC and DMAHDM composite was shown to have even greater synergistic mechanisms of action, with further reductions of CFU counts, metabolic activity, protein adsorption and polysaccharide production [38]. AgNPs are particularly effective in inhibiting *S. mutans* and *F. nucleatum*, and are also capable of long-distance bacterial killing [38]. Incorporating small particles sizes of AgNPs increases the surface area, thus achieving a strong antimicrobial function with a relatively low filler level of AgNPs without compromising the mechanical properties or aesthetics of the resin [38,41].

Despite the interdisciplinary role AgNP has in modern medicine, recent reviews have discussed the possible environmental and economic impacts of AgNPs that are synthesized either naturally (green synthesis) or chemically [55,56,57,58]. Moreover, development of AgNPs using the biological method of synthesis with the use of bacteria, fungi and plant extracts [55,56] have shown to be ecofriendlier, cost effective and energy efficient [59], with reports of greater antimicrobial activity against pathogenic bacteria [55]. All three studies that experimented with AgNPs in this review synthesized the nanoparticles through chemical reduction. As such, further research is required to examine the effects of incorporating biosynthesized AgNPs on the efficacy of DMAHDM nanocomposite.

While NACP did not exhibit any antibacterial effect and showed comparable results to commercial control composites, NACP was effective on remineralization of tooth structure and pH neutralization [1,2,3,20,37,38,39,42,44,47]. The addition of NACP enables the release of Ca and P ions to increase the pH during cariogenic challenges, thereby, preventing demineralization and facilitating remineralization [38] and thus, reducing the potential for secondary caries development. Recent studies found no significant difference between the NACP and DMAHDM + NACP composites, meaning that DMAHDM did not impact the release of ions and can therefore be incorporated at no disadvantage [1,2,38]. Furthermore, the addition of NACP in the composite resin allows for repeated recharges, facilitates ion rerelease and remineralization over a longer period of time [1]. DMAHDM maintained potent antibiofilm properties after 12 cycles of recharge and did not affect ion re-release concentrations [1,2]. The findings from Bhadila et al. (2020) indicate that the concentration of Ca and P ion releases from NACP and DMAHDM combinations surpassed the required levels for tooth remineralization [60]. These results are very promising for improving the longevity and prognosis of current restorative materials.

Xiao et al. (2017) reported synergistic remineralizing effects such as greater acid neutralization, dentine hardness and mineral growth when combining NACP and third generation PAMAM. PAMAM exhibits the ability to remineralize tooth lesions through its role as an excellent nucleation template whereby Ca and P ions are rapidly absorbed leading to remineralization [23,61,62]. PAMAM has also shown lasting dentine mineral regeneration when incorporated with recharged NACP after prolonged fluid exposure [63], which indicates successful long-term therapeutic effects in reducing demineralization and aiding in the reduction of secondary caries.

Xiao et al. (2017) suggested that the incorporation of MPC, AgNPs, MPC and PAMAM with DMAHDM yielded the maximum antibacterial and remineralization capacity. The addition of MPC and AgNPs with DMAHDM produced significant synergistic antibacterial effects, while NACP and PAMAM provided continuous ion release and combined remineralization mechanisms of action [23]. Therefore, this novel bioactive composite combination shows promising results that may adjunctively reduce the rate of secondary caries and increase the longevity of these restorations.

The findings of this systematic review suggest that incorporation of antibacterial and remineralizing biomaterials have the potential to aid in the prevention of secondary caries. However, caries is a complex multifactorial disease and other factors must be considered when determining the clinical success of composite resins. These include but are not limited to, quality of the restoration such as the presence of microgaps and patient caries risk including oral hygiene habits, salivary flow and composition, consumption of dietary sugars and exposure to fluoride [64].

The limitations of this review include medium risk of bias and in vitro conditions in all studies. Additionally, there were wide variations of comparison groups between the studies. This study only focused on antibacterial and remineralization properties and did not consider mechanical qualities during water-aging. The bacterial incubation period for samples tested for antibacterial efficacy were heterogeneous across the studies, ranging from two days in some studies to 185 days in one study. The differences in incubation protocols may have depended on the manufacturer’s instructions, pH of biofilm culture medium and that different types of bacteria required different incubation times. Therefore, it is important to note that these variations were adjusted accordingly to target specific bacterial species for different studies.

## 5. Conclusions

DMAHDM and MPC or DMAHDM and NACP were more effective than MPC or NACP alone respectively, in preventing bacterial biofilm formation, bacterial colonization and metabolic activity of biofilms. The incorporation of AgNPs was further effective on periodontal pathogens. The addition of NACP resulted in continuous ion release with the ability to recharge. The combined inclusion of NACP and PAMAM resulted in greater acid neutralization, dentine hardness, remineralization and mineral growth than NACP or PAMAM alone. The synergistic mechanisms of action of incorporating MPC, NACP, AgNPs and PAMAM with DMAHDM composite resin proposes a promising future in the development of dental materials to reduce the recurrence of secondary caries. However, we would like to reiterate that these findings emerged from in vitro studies. Well-designed randomized controlled human trials with longer follow-up periods and uniform protocols are required to provide further understanding in using antibacterial and remineralizing composite resins for improved clinical success.

## Figures and Tables

**Table 1:** Search strategy.

Serial Numbers (#)	Search Term
#1	dental composite [All Fields]
#2	composite resin [All Fields]
#3	dental nanocomposite [All Fields]
#4	dental monomer [All Fields]
#5	(#1 OR #2 OR #3 OR #4)
#6	DMAHDM [All Fields]
#7	dimethylaminohexadecyl methacrylate [All Fields]
#8	QAM [All Fields]
#9	quaternary ammonium methacrylate [All Fields]
#10	quaternary ammonium monomers [All Fields]
#11	quaternary ammonium compounds [All Fields]
#12	quaternary ammonium salts [All Fields]
#13	(#5 OR #6 OR #7 OR #8 OR #9 OR #10 OR #11 OR #12)
	Final search term: #5 AND #13

**Table 2:** Tests for antibacterial and remineralizing outcomes.

Antibacterial Outcomes	Remineralizing Outcomes
Colony-forming units (CFU): mean differences in CFU count of biofilms on experimental and control composites Lactic acid production: mean differences in lactate concentrations (mmol/L) of biofilms on experimental and control composites Metabolic activity (MTT): mean differences in optical density 450/cm^2^ of biofilms on experimental and control composites Biofilm culture medium pH: mean differences in pH of culture mediums on experimental and control composites Live/dead assay: images taken at random (qualitative variable) of bacteria on experimental and control composites Polysaccharide production: mean difference in optical density 450/cm^2^ of experimental and control composites	Calcium and phosphate ion concentrations: mean difference in calcium and phosphate ion concentrations (mmol/L) of experimental and control composites immersed in a solution Dentine hardness: mean differences in GPa (gigapascals) of root surfaces adjacent to experimental and control composites

**Table 3:** Risk of bias among the included studies.

Reference	Sample Size Calculation	Adequate Control Group	Use of Materials According to Manufacturer’s Instruction	Standardized Sample Production Process	Standardized Antibacterial/ Remineralizing Assessment	Evaluation of Antibacterial/ Remineralizing Properties by a Single Operator	Adequate Statistical Analysis	Risk of Bias *
Wu et al., 2015 [37]	No	Yes	No	Yes	Yes	No	Yes	Medium risk
Wu et al., 2015 [39]	No	Yes	No	Yes	Yes	No	Yes	Medium risk
Zhang et al., 2015 [40]	No	Yes	No	Yes	Yes	No	Yes	Medium risk
Melo et al., 2016 [41]	No	Yes	No	Yes	Yes	No	Yes	Medium risk
Wang et al., 2016 [42]	No	Yes	No	Yes	Yes	No	Yes	Medium risk
Wang et al., 2016 [43]	No	Yes	No	Yes	Yes	No	Yes	Medium risk
Xie et al., 2016 [44]	No	Yes	No	Yes	Yes	No	Yes	Medium risk
Zhang et al., 2016 [20]	No	Yes	No	Yes	Yes	No	Yes	Medium risk
Xiao et al., 2017 [23]	No	Yes	No	Yes	Yes	No	Yes	Medium risk
Zhang et al., 2017 [45]	No	Yes	No	Yes	Yes	No	Yes	Medium risk
Al-Dulaijan et al., 2018 [1]	No	Yes	No	Yes	Yes	No	Yes	Medium risk
Wang et al., 2019 [46]	No	Yes	No	Yes	Yes	No	Yes	Medium risk
Xiao et al., 2019 [38]	No	Yes	No	Yes	Yes	No	Yes	Medium risk
Bhadila et al., 2020 [2]	No	Yes	No	Yes	Yes	No	Yes	Medium risk
Zhou et al., 2020 [47]	No	Yes	No	Yes	Yes	No	Yes	Medium risk

* Risk of bias criteria; 1–3 “Yes” = high risk; 4–5 “Yes” = medium risk; 6–7 “Yes” = low risk.

**Table 4:** Antibacterial results.

Reference	Biomaterial Combinations Used and Comparison Group/s	Microorganisms Tested	Time Points Assessed	Methodology Used to Assess Antibacterial Effectiveness	Results Summary
Wu et al., 2015 [37]	(1–5) Five variants with 10% DMAHDM + 20% NACP + 0%, 2.5%, 5%, 7.5% or 10%, respectively, of microcapsules of formaldehyde and urea Comparison group: (6) 20% NACP	Total microorganism Total streptococci *Streptococcus mutans*	2 days	Live/dead assay	Biofilm was primarily alive in (6) (1) showed mostly dead bacteria.
				Metabolic assay (MTT)	Metabolic activity of (1) was reduced by 96% compared to (6)
				Production of Lactic Acid	(1) reduced lactic acid production by 99% compared to (6)
				CFUc counts	(1) reduced the biofilm CFU by 3–4 times compared to (6)
Wu et al., 2015 [39]	(1–4) Four variants with 20% NACP and DMAHDM mass fraction of 0.75%, 1.5%, 2.25% and 3%, respectively Comparison group: (5) 20% NACP	Total microorganisms Total streptococci *S. mutans*	2 days	Live/dead assay	Biofilm was primarily alive in (5). Amounts of dead bacteria increased with increase in the mass fraction of DMAHDM
				Metabolic assay (MTT)	Metabolic activity—(4) was 96% lower than (5)
				Production of Lactic Acid	Increasing DMAHDM mass fraction caused a monotonic decrease in the production of lactic acid
				CFUc counts	Antibacterial activity increased and CFU decreased with increase in the mass fraction of DMAHDM
Zhang et al., 2015 [40]	(1) 1.5% DMAHDM (2) 3% MPC (3) 1.5% DMAHDM + 3% MPC Comparison groups: (4) 0% DMAHDM + 0% MPC (5) Heliomolar commercial composite	Total microorganisms Total streptococci *S. mutans*	2 days	Live/dead assay	(1) demonstrated lower bacterial adhesion but most bacteria were alive (1) showed high amounts of dead bacteria
				Metabolic assay (MTT)	(3) had the least metabolic activity and higher reduction in biofilm growth compared to (1) or (2)
				Production of Lactic Acid	(3) had the least lactic acid production
				CFU counts	(3) reduced CFU counts by >3 logs compared to (4) or (5) and had much less biofilm CFU than (1) or (2)
				Protein adsorption	(3) had less protein adsorption compared to controls than (1) or (2)
Melo et al., 2016 [41]	(1) 5% DMAHDM + 0.1% AgNPs + 30% NACP Comparison group: (2) 0% DMAHDM + 0% AgNPs + 0% NACP	Did not specify	7 days	Live/dead assay	Dental materials containing multiagents resulted in compromised bacteria at tooth-composite interface
Wang et al., 2016 [42]	(1) 3% DMAHDM + 20% NACP Comparison groups: (2) 20% NACP (3) Heliomolar commercial composite	*P. gingivalis,* *P. intermedia,* *Prevotella nigrescens, * *A. actinomycetemcomitans,* *F. nucleatum,* *Enterococcus faecalis*	2 days	Live/dead assay	(1) mainly had dead bacteria while (2) and (3) had primarily live bacteria
				CFU counts	(1) CFU reduction differed between the bacterial species differently, few by <3 log while others by >3 log
				Crystal violet biofilm biomass assay	(1) had a significantly decreased biomass value compared to (2) and (3)
				Polysaccharide production	(1) had greatly reduced polysaccharide production for all six species compared to (2) and (3)
Wang et al., 2016 [43]	(1) 3% DMAHDM (2) 3% MPC (3) 3% DMAHDM + 3% MPC ^b^ Comparison groups: (4) 0% DMAHDM + 0% MPC (5) Heliomolar commercial composite	*P. gingivalis,* *P. intermedia,* *A. actinomycetemcomitans,* *F. nucleatum*	2 days	Live/dead assay	(2) reduced the adhesion of bacteria, (3) demonstrated mostly dead bacteria
				Metabolic activity (MTT)	(3) presented lower biofilm metabolic activity on all the tested periodontal pathogens compared to (4) and (5)
				CFU counts	Addition of DMAHDM or MPC independently into the composite decreased the CFU
				Protein adsorption	(1) had no effect on protein adsorption (2) substantially decreased the protein adsorption by one log compared to (4) and (5)
				Polysaccharide production	(3) had much less polysaccharide production compared to (4) and (5)
Xie et al., 2016 [44]	(1) 30% NACP + 3% MPC (2) 30% NACP + 3% MPC + 1.5% DMAHDM (3) 30% NACP + 3% MPC + 3% DMAHDM Comparison groups: (4) 30% NACP (5) Heliomolar commercial composite	Total microorganisms Total streptococci *S. mutans*	2 days (with 2-day biofilm) and 4 days (pH required 72 h of incubation)	Live/dead assay	(4) and (5) were completely covered by live bacteria. Bacterial adhesion was reduced by MPC, DMAHDM produced an antibacterial effect. (3) had the most dead bacteria followed by (2) and (1)
				Metabolic assay (MTT)	Metabolic activity of biofilms of (3) < (2) < (1) (3) had the lowest metabolic activity of biofilms among all
				CFU counts	(3) had the least biofilm CFU, count reduced by 3 logs compared to (4) and (5).
				Protein adsorption	(1) had protein adsorption one log less than (5); (2) and (3) had no effect on the protein adsorption
				pH of biofilm culture medium	(3) maintained a pH above 6.5.
Zhang et al., 2016 [20]	(1–5) Five variants with 20% NACP with QAM CL of 3, 6, 12, 16 and 18, respectively Comparison groups: (6) 20% NACP (7) Renamel Microfill commercial composite	Total microorganisms Total streptococci *S. mutans*	30 days	Live/dead assay	(6) and (7) were covered by live bacteria. Dead bacteria increased progressively from CL3 up to CL16 with maximum antibacterial potency at CL16 before decreasing in potency at CL18 as indicated by some live bacteria.
				Metabolic assay (MTT)	Metabolic activity of biofilms decreased with increase in CL from 3 to 16. CL16 had maximum reduction on metabolic activity, which remained constant with any increase in CL
				Production of Lactic Acid	The biofilms on (6) and (7) produced the most acid. Acid production capacity of biofilm increased with an increase in CL from 3 to 16 CL16 minimized lactic acid production by 10-fold compared to (6) and (7)
				CFU counts	CFU counts decreased with an increase in the CL from 3 to 16. Antibacterial activity was strongest at CL16, which lowered at C18. CL16 reduced all three CFU counts by 2 logs compared to (6) and (7)
Zhang et al., 2017 [45]	(1) 1.5% DMAHDM (2) 3% MPC (3) 1.5% DMAHDM + 3% MPC Comparison group: (4) Heliomolar commercial composite	Total microorganisms Total streptococci *S. mutans*	185 days	Live/dead assay	(3) had high levels of dead bacteria and lower bacterial attachment. Protein-repellent and anti-biofilm activities remained same from day 1 to 180
				Metabolic assay (MTT)	(1) and (2) showed higher reduction of biofilm viability than (4) (3) had the least metabolic activity Antibacterial function remained the same from day 1 to 180, being unimpacted by water-aging
				Production of Lactic Acid	(3) had the least lactic acid production
				CFU counts	(1) and (2) decreased the CFU compared to (4). (3) had greater antibacterial properties compared to (1) and (2) and was nearly 3 logs lower than (4), both at 1 day and 180 days of water-aging (*p* < 0.05).
				Protein adsorption	MPC greatly inhibited protein adsorption with no difference between 1 day and 180 days. (3) had the same protein adsorption as (2) (*p* > 0.1), which was about one tenth that of (4) and (1) (*p* < 0.05).
Al-Dulaijan et al., 2018 [1]	(1) 20% NACP + 3% DMAHDM (2) 20% NACP Comparison group: (3) Heliomolar commercial composite	Total microorganisms Total streptococci *S. mutans*	2 days	Live/dead assay	(1) had much less live bacteria compared to (2) and (3).
				Metabolic assay (MTT)	(1) greatly decreased the metabolic activity of the biofilms compared to (2) and (3) (*p* < 0.05). (2) had similar metabolic activity to (3) indicating that NACP had little effect on biofilm viability.
				Production of Lactic Acid	(1) had the least lactic acid production.
				CFU counts	(1) decreased all three CFU counts by 3–4 logs compared to (2) and (3).
Wang et al., 2019 [46]	(1) 3% MPC + 20% NACP (2) 3% DMAHDM + 20% NACP (3) 3% DMAHDM + 3% MPC + 20% NACP Comparison groups: (4) 20% NACP (5) Heliomolar commercial composite	(1) Biofilm with one species: *P. gingivalis* (2) Biofilm with three species: *P. gingivalis, S. gordonii* and *F. nucleatum* (3) Biofilm with six species: *P. gingivalis, S. gordonii, F. nucleatum, A. naeslundii, P. intermedia* and *A. actinomycetemcomitans* (4) Nine-species biofilm: *P. gingivalis, S. gordonii, F. nucleatum, A. naeslundii, P. intermedia, A. actinomycetemcomitans, P. nigrescens, Tannerella forsythia* and *Parvimonas micra*	4 days	Live/dead assay	(2) had large quantity of dead bacteria, (1) showed lower bacterial adhesion. (3) had large quantity of dead bacteria but lower bacterial adhesion that (4) and (5) which were largely covered by live bacteria
				Metabolic assay (MTT)	(1) and (2) reduced the metabolic activity greatly compared to (3) Killing power of DMAHDM decreased with increase in the number of species in the biofilm (3) had stronger killing efficacy on all biofilm types
				CFU	(3) had higher reduction of CFU than (1) and (2), by >3 log on all four biofilm types
				Protein adsorption	DMAHDM demonstrated no effect on protein adsorption (1) decreased the protein adsorption by approximately 1 log, compared to (2) and (5)
				Polysaccharide production	Single species biofilms produced less polysaccharides than multi species biofilms (1) and (2) decreased the amount of polysaccharide produced by biofilms, (3) showed least production in all the biofilm types
Xiao et al., 2019 [38]	(1) 30% NACP + 3% MPC + 3% DMAHDM (2) 30% NACP + 3% MPC + 3% DMAHDM + 0.12% AgNPs ^a,c^ Comparison groups: (3) 30% NACP (4) Renamel Microfill commercial composite	(1) *P. gingivalis* (2) *A. actinomycetemcomitans* (3) *F. nucleatum*	2 days	Live/dead assay	(1) and (2) had much less biofilms, with mostly dead bacteria compared to (3) and (4), which were mostly covered by live bacteria.
				Metabolic assay (MTT)	Metabolic activity of (1) and (2) lower than (3) and (4) (2) showed lower biofilm metabolic activity for all three bacteria species than (1)
				Production of Lactic Acid	(1) had significantly decreased CFU counts for all three species, (2) showed the lowest CFU. (1) reduced the CFU counts by 4 logs on *P. gingivalis* and *A. actinomycetemcomitans*, while (2) reduced the CFU counts by 5 log. (1) and (2) reduced the CFU counts for F. nucleatum, by 3 and 5 logs, respectively.
				Protein adsorption	(1) and (2) decreased protein adsorption, it was e tenth of (3) and (4)
				Polysaccharide production	(1) had much less polysaccharide production than (3) and (4 The lowest production of polysaccharides from biofilms was caused by (2)
Bhadila et al., 2020 [2]	(1) 20% NACP (2) 3% DMAHDM + 20% NACP Comparison group: (3) Heliomolar commercial composite	*S. mutans*	2 days	Live/dead assay	(2) had primarily dead bacteria compared to (1) and (3) which were primarily covered by live bacteria.
				Production of Lactic Acid	(2) caused lowest production of lactic acid production from biofilms than (1) and (3)
				CFU counts	(2) showed a CFU reduction of 3–4 logs less than (1) and (3).
Zhou et al., 2020 [47]	(1) 30% NACP (2) 3% DMAHDM (3) 30% NACP + 3% DMAHDM Comparison groups: (4) 0% NACP + 0% DMAHDM (5) Heliomolar commercial composite	*S. mutans* *L. acidophilus* *C. albicans* Multi-species	2 days	Live/dead assay	(2) had substantial dead bacteria for all species tested while (1), (4) and (5) were covered with live bacteria.
				Metabolic assay (MTT)	(2) reduced the metabolic activity of biofilms significantly
				Production of Lactic Acid	(1), (4) and (5) showed higher lactic acid production from *S. mutans* and polymicrobial biofilms while (2) and (3) inhibited. All materials produced lower levels of lactic acid from *L. acidophilus* and *C. albicans*
				CFU counts	(2) and (3) greatly reduced *S. mutans* and *C. albicans* CFU levels by 5 and 3 logs respectively
				Polysaccharide production	(2) and (3) inhibited production of extracellular matrix from the bacterial associated with root caries

DMAHDM—Dimethylaminohexadecyl Methacrylate. NACP—Nanoparticles of Amorphous Calcium Phosphate. CFU—Colony-Forming Units. MPC—2-Methacryloyloxyethyl Phosphorylcholine. AgNPs—Silver Nanoparticles. ^a^ Other composite groups excluded in antibacterial tests due to reduced mechanical properties. ^b^ Other composite groups excluded in antibacterial tests due to reduced protein repellency QAM (quaternary ammonium methacrylate) CL (chain length). ^c^ Selected as the experimental group due to superior mechanical properties and a previous study showed higher levels of AgNPs produces greater antibacterial effect.

**Table 5:** Remineralization results.

Reference	Biomaterial Combinations and Comparison Group/s	Time Points Assessed	Methodology Used to Assess Remineralization	Results Summary
Zhang et al., 2016 [20]	(1) 20% NACP with QAM CL16 ^a^ Comparison group: (2) 20% NACP	1, 3, 7, 14, 21 and 28 days	Release of Ca and P ions	(1) released lower levels of Ca and P ions than (2)
Xiao et al., 2017 [23]	(1) 0.12% AgNPs + 3% MPC + 3% DMAHDM + 30% NACP (2) 0.12% AgNPs + 3% MPC + 3% DMAHDM + 30% NACP + PAMAM Comparison groups: (3) Demineralized root dentine specimen (4) Demineralized root dentine specimen + PAMAM	1, 3, 5, 7, 10, 14 and 21 days	Concentration of Ca and P ions	(1) and (2) demonstrated greater concentrations of Ca and P concentrations than PAMAM and control groups
			Acid neutralization	(1) and (2) had greater acid neutralization than PAMAM and comparison groups.
			Dentine hardness	(2) had the greatest dentine hardness, remineralization and mineral growth.
			SEMexamination	(2) had the greatest remineralization and mineral growth.
Al-Dulaijan et al., 2018 [1]	(1) 20% NACP + 3% DMAHDM (2) 20% NACP Comparison group: (3) Heliomolar commercial composite	1, 3, 5, 7, 14, 21, 28, 35, 42, 49, 56, 63 and 70 days	Concentration of Ca and P ions	No differences in concentrations of Ca and P ions, their recharge and re-release between (1) and (2) groups
		1, 2, 3, 5, 9, 11 and 14 days	Recharge and rerelease of Ca and P	Specimens could release the ions for 42 days after one charge
Bhadila et al., 2020 [2]	(1) 20% NACP (2) 3% DMAHDM + 20% NACP Comparison group: (3) Heliomolar commercial composite	1, 3, 5, 7, 14, 21, 28, 35, 42, 49, 56, 63 and 70 days	Release of Ca and P ions	No significant difference in Ca and P ion release between (1) and (2)
		1, 3, 5, 7, 9 and 14 days	Recharge and rerelease of Ca and P	Both composites showed increasing ion concentration with time, and release continued after each recharge.
Zhou et al., 2020 [47]	(1) 30% NACP (2) 3% DMAHDM (3) 30% NACP + 3% DMAHDM Comparison groups: (4) 0% NACP+ 0% DMAHDM (5) Heliomolar commercial composite	1, 3, 7, 14, 21, 28, 35, 42, 49, 54, 63 and 70 days	Release of Ca and P ions	No difference in release of Ca and P ions between (1) and (3) Lower pH increased ion release
			Dentine hardness	(3) caused the highest dentine hardness, which was twice more than that of controls.

NACP—Nanoparticles of Amorphous Calcium Phosphate. QAM—Quaternary Ammonium Methacrylate. CL—Chain Length. Ca—Calcium. P—Phosphate. AgNPs—Silver Nanoparticles. MPC—2-Methacryloyloxyethyl Phosphorylcholine. DMAHDM—Dimethylaminohexadecyl Methacrylate. PAMAM—Polyamidoamine dendrimer. SEM—Scanning Electron Microscopy. ^a^ Other composite groups excluded in remineralization tests as CL 16 exhibited the strongest antibacterial activity.

## Data Availability

The data presented in this study are available on request from the Corresponding author.

## Supplementary Materials

The following are available online at https://www.mdpi.com/article/10.3390/ma14071688/s1: Table S1: Background characteristics of the included studies.
