# Supplementary material for: The Antibacterial and Remineralizing Effects of Biomaterials Combined with DMAHDM Nanocomposite: A Systematic Review
Source: Materials (Basel). 2021 Mar 30;14(7):1688. doi: 10.3390/ma14071688 (PMC8037094; doi:10.3390/ma14071688)
Supplement: Supplementary file 1 [file materials-14-01688-s001.pdf]

materials

MDPI

Supplementary Materials

The Antibacterial and Remineralizing Effects of Biomaterials Combined with DMAHDM Nanocomposite: A Systematic Review

Alison Clarin [1], Daphne Ho [1], Jana Soong [1], Cheryl Looi [1], Deepak Samuel Ipe [1,2] and Santosh Kumar Tadakamadla [1,2,*]

[1] School of Dentistry and Oral Health, Griffith University, Gold Coast 4217, Australia; alison.clarin@griffithuni.edu.au (A.C.); daphne.ho@griffithuni.edu.au (D.H.); jana.soong@griffithuni.edu.au (J.S.); cheryl.looi@griffithuni.edu.au (C.L.); deepu313@gmail.com (D.S.I.)
[2] Menzies Health Institute Queensland, Gold Coast 4217, Australia
* Correspondence: santoshkumar.tadakamadla@griffithuni.edu.au; Tel.: +61-756780153

Table S1. Background characteristics of the included studies

Reference	Sample Size/s	Biomaterial Combinations Used and Comparison Group/s	Inoculum Used and Collection Method
Wu et al. 2015 [1]	Mechanical testing – 36 disks (n = 6)<br>MTT metabolic assay – 36 disks (n = 6)<br>Lactic acid production – 36 disks (n = 6)<br>Live/dead assay – 36 images (n = 6)<br>CFU counts – 108 disks (n = 6, m = 3)	(1–5) Five variants with BisGMA-TEGDMA + 10% DMAHDM + 20% NACP + 35% glass + 0%, 2.5%, 5%, 7.5% or 10%, respectively, of microcapsules of formaldehyde and urea<br><br>Comparison group:<br>(6) BisGMA-TEGDMA + 20% NACP + 35% glass	-Saliva collected from 10 adult donors, sterile glycerol used for dilution, placed in the growth medium as inoculum.
Wu et al. 2020 [2]	Mechanical testing – 30 disks (n = 6)<br>MTT metabolic assay – 30 disks (n = 6)<br>Lactic acid production – 30 disks (n = 6)<br>Live/dead assay – 90 images (n = 6, m = 3)<br>CFU counts – 90 disks (n = 6, m = 3)	(1–4) Four variants with 20% NACP and DMAHDM mass fraction of 0.75%, 1.5%, 2.25% and 3%, respectively<br><br>Comparison group:<br>(5) BisGMA-TEGDMA resin + 50% glass + 20% NACP	- Followed same methodology as previous study [3]
Zhang et al. 2015 [4]	Mechanical testing – 54 disks (n = 6)<br>MTT metabolic assay – 30 disks (n = 6)<br>Lactic acid production – 30 disks (n = 6)<br>Live/dead assay – 90 images (n = 6, m = 3)<br>CFU counts – 90 disks (n = 6, m = 3)	(1–3) Three variants with DMAHDM concentrations 1.5%, 2.25% and 3% without MPC, respectively<br><br>(5–7) Three variants with DMAHDM concentrations 0%, 1.5%, 2.25%	- Followed same methodology as previous study [3]

		and 3% along with 3% MPC, respectively<br><br>Comparison groups: (8) BisGMA-TEGDMA + 70% glass<br><br>(9) Heliomolar commercial composite	
Melo et al. 2016 [5]	Live/dead assay – 15 disks (n = 15)<br>(total images not specified)	(1) BisGMA-TEGDMA + 5% DMAHDM + 0.1% AgNPs + 30% NACP<br><br>Comparison group:<br>(2) BisGMA-TEGDMA	- Saliva inoculum used, obtained from adult volunteers.<br>- Biofilm grown on sucrose (0.2%) enriched Nutrient medium.
Wang et al. 2016 [6]	Mechanical testing – 18 disks (n = 6)<br>Live/dead assay – 270 images (5 images per specimen, n = 3, m = 6)<br>CFU counts – 108 disks (n = 6, m = 6)<br>Biofilm biomass – 108 disks (n = 6, m = 6)<br>Polysaccharide production – 108 disks (n = 6, m = 6)	(1) EBPM+3%DMAHDM+20%NACP+50%glass<br><br>Comparison groups:<br>(2) EBPM+20%NACP+50% glass<br>(3) Heliomolar commercial composite	- Saliva collected from 15 adult donors.<br>- Composite disks in a 24-well plate immersed in sterile saliva for 2 hrs at 37°C.
Wang et al. 2016 [7]	Mechanical testing – 54 disks (n = 6)<br>Water sorption – 45 disks (n = 5);<br>Protein adsorption – 30 disks (n = 6);<br>Live/dead assay – 300 images (3 images per specimen, n = 5, m = 4)<br>MTT metabolic assay – 120 disks (n = 6, m = 4)<br>CFU counts – 120 disks (n = 6, m = 4)<br>Polysaccharide production – 120 disks (n = 6, m = 4)	(1-4) Four variants with EBPM, 20% NACP and MPC concentrations 0%, 1.5%, 3% and 4.5% without DMAHDM, respectively<br>(5-8) Four variants with EBPM, 20% NACP and MPC concentrations 0%, 1.5%, 3% and 4.5% along with 3% DMAHDM, respectively<br><br>Comparison group:<br>(9) Heliomolar commercial composite	- Followed same methodology as previous study [8]
Xie et al. 2016 [9]	Mechanical testing – 60 disks (n = 6)<br>Protein absorption – 30 disks (n = 6)<br>MTT assay – 90 disks (n = 6, m = 3)<br>Live/dead assay – 90 images (3 images per specimen, n = 6)<br>CFU counts – 90 disks (n = 6, m = 3)	(1) EBPM + 35% glass + 30% NACP + 3% MPC<br>(2) EBPM + 35% glass + 30% NACP + 3% MPC + 1.5% DMAHDM<br>(3) EBPM + 35% glass + 30% NACP + 3% MPC + 3% DMAHDM<br><br>Comparison groups:<br>(4) EBPM + 35% glass + 30% NACP	- Followed same methodology as previous study [3]

	pH of biofilm – 30 culture mediums (n = 6)	(5) Heliomolar commercial composite	
Zhang et al. 2016 [10]	Mechanical testing – 21 disks (n = 6)<br>Live/dead assay – 84 images (4 images per specimen, n = 3)<br>MTT metabolic assay – 42 disks (n = 6)<br>Lactic acid production – 42 disks (n = 6)<br>CFU counts – 147 disks (n = 7, m = 3)<br>Ca and P ion release – 24 disks (3 pH levels per group, n = 4)	(1-5) Five variants with BisGMA-TEGDMA + 20% NACP + 50% glass with QAM CL of 3, 6, 12, 16 and 18, respectively<br><br>Comparison groups:<br>(6) BisGMA-TEGDMA + 20% NACP + 50% glass<br>(7) Renamel Microfill commercial composite	- Followed same methodology as previous study [3]
Xiao et al. 2017 [11]	Mechanical testing – 24 disks (n = 6)<br>Ca and P ion release – 48 specimens (2 pH levels per group, n = 6)<br>Acid neutralisation – 48 specimens (2 pH levels per group, n = 6)<br>Dentine hardness – 36 specimens (n = 6)<br>SEM examination – 24 specimens (n = 6)	(1) EBPM-AgNPs+3%MPC+3% DMAHDM+30% NACP+35% glass<br>(2) EBPM-AgNPs+3% MPC+3% DMAHDM+30% NACP+35%glass+PAMAM<br><br>Comparison groups:<br>(3) Demineralised root dentine specimena<br>(4) Demineralised root dentine specimen + PAMAMa<br>(5) EBPM + 30% NACP + 35% glassb<br>(6) Heliomolar commercial compositeb<br>(7) Renamel Microfill commercial compositeb<br>(8) Sound dentine specimemc<br>(9) Acid-etched dentine specimenc	- Permanent premolars of humans that were single rooted and caries-free were disinfected and stored in distilled water.<br>- Root dentine specimens were acid-etched to create demineralization and divided into 4 groups.<br>- All specimens were immersed in artificial saliva at neutral pH for23 hours, followed by one hour immersion in lactic acid solution (pH 4.0) each day for 21 days.
Zhang et al. 2017 [12]	Mechanical testing – 24 disks (n = 6)<br>Protein adsorption – 24 disks (n = 6)<br>Live/dead assay – 24 images (2 images per specimen, n = 6)<br>Metabolic activity – 30 disks (n = 6)<br>Lactic acid production - 30 disks (n = 6)<br>CFU counts – 48 disks (n = 4, m = 3)	(1) BisGMA-TEGDMA + 70% glass + 3% MPC<br>(2) BisGMA-TEGDMA + 70% glass + 1.5% DMAHDM<br>(3) BisGMA-TEGDMA + 70% glass + 3% MPC + 1.5% DMAHDM<br><br>Comparison group:<br>(4) Heliomolar commercial composite	- Saliva collected from 10 adult donors and an oral plaque microcosm model used to incubate biofilms on the polymeric composite.
Al-Dulaijan et al. 2018 [13]	Mechanical testing – 18 disks (n = 6)<br>Live/dead assay – 54 images (18 images per specimen, n = 6)<br>MTT metabolic activity – 18 disks (n = 6)<br>Lactic acid production – 18 disks (n = 6)<br>CFU counts – 54 disks (n = 6, m = 3)<br>Ca & P ions:	(1) EBPM+20%NACP+50%glass+3%DMAHDM<br>(2) EBPM+20%NACP+50%glass<br><br>Comparison group:<br>(3) Heliomolar commercial composite	- Saliva collected from 10 adult donors.<br>- Composite disks in a 24-well plate incubated in 5% CO_2 for 8hrs at 37°C.

	Initial release – 12 disks (n = 6); Recharge & re-release – 6 disks (n = 3).		
Wang et al. 2019 [14]	Surface roughness – 30 disks (n = 6)<br>Composite surface charge density – 30 disks (n = 6)<br>Protein adsorption – 30 disks (n = 6)<br>Live/dead assay – 90 images (18 images per specimen, n = 6)<br>CFU counts – 120 disks (n = 6, m = 4)<br>Metabolic assay – 120 disks (n = 6, m = 4)<br>Polysaccharide production – 120 disks (n = 6, m = 4)	(1) EBPM+3%MPC+20%NACP+50% glass<br>(2) EBPM+3%DMAHDM+20%NACP+50% glass<br>(3) EBPM+3%DMAHDM+3%MPC+20%NACP+50% glass<br><br>Comparison groups:<br>(4) EBPM+20%NACP+50%glass<br>(5) Heliomolar commercial composite	- Saliva collected from 15 adult donors was centrifuged and sterilized.<br>- Composite disks in a 24-well plate immersed in sterile saliva for 2 hrs at 37°C.
Xiao et al. 2019 [15]	Mechanical testing – 42 disks (n = 6)<br>Protein adsorption – 24 disks (n = 6)<br>Live/dead assay – 180 images (15 images per specimen, n = 3, m = 3)<br>MTT metabolic activity – 72 disks (n = 6, m=3)<br>Polysaccharide production – 72 disks (n = 6, m = 3)<br>CFU counts – 72 disks (n = 6, m = 3)	(1-4) Four variants with EBPM+30%NACP+3%MPC+3%DMAHDM +35%glass and AgNPs concentrations 0%, 0.08%, 0.12% and 0.16%, respectively<br><br>Comparison groups:<br>(5) EBPM+30%NACP+35% glass<br>(6) Renamel Microfill commercial composite<br>(7) Heliomolar commercial composite	- Saliva collected from 12 adult donors.<br>- Composite disks in a 24-well plate immersed in sterile saliva for 2 hrs at 37°C.
Bhadila et al. 2020 [16]	CFU counts – 15 disks (n = 5)<br>Lactic acid production – 15 disks (n = 5)<br>Ca & P ions:<br>Initial release – 24 disks(n = 8); Recharge & re-release – 12 disks (n = 4)	(1) EBPM+20%NACP+50% glass<br>(2) EBPM+3%DMAHDM+20%NACP+50% glass<br><br>Comparison group:<br>(3) Heliomolar commercial composite	- Composite disks in a 24-well plate immersed in sterile saliva for 16 hrs at 37°C.
Zhou et al. 2020 [17]	Mechanical testing - 30 disks (n = 6)<br>Live/dead staining - 180 images (3 images per specimen, n = 3, m = 4)<br>MTT metabolic assay – 120 disks (n = 6, m = 4)<br>Lactic acid production – 120 disks (n = 6, m = 4)	(1) BisGMA-TEGDMA+30%NACP+35% glass<br>(2) BisGMA-TEGDMA+3%DMAHDM+65% glass<br>(3) BisGMA-TEGDMA+30%NACP+3%DMAHDM+35% glass<br><br>Comparison groups:<br>(4) BisGMA-TEGDMA + 65% glass<br>(5) Heliomolar commercial composite<br>(6) Sound dentine specimen	- 200 mL of water (deionized) was used to submerge composite disks and were then centrifuged.<br>- Disks sterilized in ethylene oxide and degassed for 1 week.<br>- 30 bovine teeth were used to make root dentine disks that were etched and randomly bonded to a composite specimen.

Polysaccharide production – 120 disks (n = 6, m = 4)	
Dentine hardness – 30 root dentine disks (n = 6, m = 1)	

Number of specimens per composite groupCFU (colony-forming units)
m (number of microorganism groups tested)
BisGMA-TEGDMA - Bisphenol A-Glycidyl MethAcrylate and TriEthylene Glycol DiMethAcrylate mixed at 1:1(mass) ratio and rendered light-curable with addition of 0.2% camphorquinone and 0.8% ethyl 4-N,Ndimethylaminobenzoate
DMAHDM - DiMethylAminoHexaDecyl Methacrylate
NACP - Nanoparticles of Amorphous Calcium Phosphate
MPC - 2-Methacryloyloxyethyl PhosphorylCholine
AgNPs- Silver nanoparticles
EBPM- Ethoxylated BisPhenol A DiMethAcrylate (EBPADMA) and PyroMellitic GlycerolDiMethacrylate (PMGDM) mixed at a 1:1(mass) ratio and rendered light-curable with addition of 0.2% camphorquinone and 0.8% ethyl 4-N,N-diethylaminobenzoateCa (calcium)
P (phosphate)
QAM - Quaternary Ammonium Methacrylate
CL - Chain Length
SEM - Scanning Electron Microscoy
PAMAM – PolyAMidoAMine dendrimer
a Comparison group/s for all remineralizing tests
b Comparison group/s for mechanical tests only
c Comparison group/s only included for root dentine hardness test

References

1. Wu, J.; Weir, M.D.; Melo, M.A.; Xu, H.H. Development of novel self-healing and antibacterial dental composite containing calcium phosphate nanoparticles. *Journal of dentistry* **2015**, *43*, 317–326, doi:10.1016/j.jdent.2015.01.009.
2. Wu, J.; Zhou, H.; Weir, M.D.; Melo, M.A.; Levine, E.D.; Xu, H.H. Effect of dimethylaminohexadecyl methacrylate mass fraction on fracture toughness and antibacterial properties of CaP nanocomposite. *Journal of dentistry* **2015**, *43*, 1539–1546, doi:10.1016/j.jdent.2015.09.004.
3. Zhou, H.; Weir, M.D.; Antonucci, J.M.; Schumacher, G.E.; Zhou, X.D.; Xu, H.H.K. Evaluation of three-dimensional biofilms on antibacterial bonding agents containing novel quaternary ammonium methacrylates. *International Journal of Oral Science* **2014**, *6*, 77–86, doi:10.1038/ijos.2014.18.
4. Zhang, N.; Ma, J.; Melo, M.A.; Weir, M.D.; Bai, Y.; Xu, H.H. Protein-repellent and antibacterial dental composite to inhibit biofilms and caries. *Journal of dentistry* **2015**, *43*, 225–234, doi:10.1016/j.jdent.2014.11.008.
5. Melo, M.A.; Orrego, S.; Weir, M.D.; Xu, H.H.; Arola, D.D. Designing Multiagent Dental Materials for Enhanced Resistance to Biofilm Damage at the Bonded Interface. *ACS applied materials & interfaces* **2016**, *8*, 11779–11787, doi:10.1021/acsami.6b01923.

6. Wang, L.; Melo, M.A.; Weir, M.D.; Xie, X.; Reynolds, M.A.; Xu, H.H. Novel bioactive nanocomposite for Class-V restorations to inhibit periodontitis-related pathogens. *Dental materials : official publication of the Academy of Dental Materials* **2016**, *32*, e351-e361, doi:10.1016/j.dental.2016.09.023.
7. Wang, L.; Xie, X.; Imazato, S.; Weir, M.D.; Reynolds, M.A.; Xu, H.H. A protein-repellent and antibacterial nanocomposite for Class-V restorations to inhibit periodontitis-related pathogens. *Materials science & engineering. C, Materials for biological applications* **2016**, *67*, 702–710, doi:10.1016/j.msec.2016.05.080.
8. Li, F.; Weir, M.D.; Chen, J.; Xu, H.H. Effect of charge density of bonding agent containing a new quaternary ammonium methacrylate on antibacterial and bonding properties. *Dental materials : official publication of the Academy of Dental Materials* **2014**, *30*, 433–441, doi:10.1016/j.dental.2014.01.002.
9. Xie, X.; Wang, L.; Xing, D.; Arola, D.D.; Weir, M.D.; Bai, Y.; Xu, H.H. Protein-repellent and antibacterial functions of a calcium phosphate rechargeable nanocomposite. *Journal of dentistry* **2016**, *52*, 15–22, doi:10.1016/j.jdent.2016.06.003.
10. Zhang, K.; Cheng, L.; Weir, M.D.; Bai, Y.X.; Xu, H.H. Effects of quaternary ammonium chain length on the antibacterial and remineralizing effects of a calcium phosphate nanocomposite. *International journal of oral science* **2016**, *8*, 45–53, doi:10.1038/ijos.2015.33.
11. Xiao, S.; Liang, K.; Weir, M.D.; Cheng, L.; Liu, H.; Zhou, X.; Ding, Y.; Xu, H.H.K. Combining Bioactive Multifunctional Dental Composite with PAMAM for Root Dentin Remineralization. *Materials (Basel, Switzerland)* **2017**, *10*, doi:10.3390/ma10010089.
12. Zhang, N.; Zhang, K.; Melo, M.A.; Weir, M.D.; Xu, D.J.; Bai, Y.; Xu, H.H. Effects of Long-Term Water-Aging on Novel Anti-Biofilm and Protein-Repellent Dental Composite. *Int J Mol Sci* **2017**, *18*, doi:10.3390/ijms18010186.
13. Al-Dulaijan, Y.A.; Cheng, L.; Weir, M.D.; Melo, M.A.S.; Liu, H.; Oates, T.W.; Wang, L.; Xu, H.H.K. Novel rechargeable calcium phosphate nanocomposite with antibacterial activity to suppress biofilm acids and dental caries. *Journal of dentistry* **2018**, *72*, 44–52, doi:10.1016/j.jdent.2018.03.003.
14. Wang, L.; Xie, X.; Qi, M.; Weir, M.D.; Reynolds, M.A.; Li, C.; Zhou, C.; Xu, H.H.K. Effects of single species versus multispecies periodontal biofilms on the antibacterial efficacy of a novel bioactive Class-V nanocomposite. *Dental materials : official publication of the Academy of Dental Materials* **2019**, *35*, 847–861, doi:10.1016/j.dental.2019.02.030.
15. Xiao, S.; Wang, H.; Liang, K.; Tay, F.; Weir, M.D.; Melo, M.A.S.; Wang, L.; Wu, Y.; Oates, T.W.; Ding, Y., et al. Novel multifunctional nanocomposite for root caries restorations to inhibit periodontitis-related pathogens. *Journal of dentistry* **2019**, *81*, 17–26, doi:10.1016/j.jdent.2018.12.001.
16. Bhadila, G.; Baras, B.H.; Weir, M.D.; Wang, H.; Melo, M.A.S.; Hack, G.D.; Bai, Y.; Xu, H.H.K. Novel antibacterial calcium phosphate nanocomposite with long-term ion recharge and re-release to inhibit caries. *Dent Mater J* **2020**, 4, 678–689, doi:10.4012/dmj.2019-203.
17. Zhou, W.; Zhou, X.; Huang, X.; Zhu, C.; Weir, M.D.; Melo, M.A.S.; Bonavente, A.; Lynch, C.D.; Imazato, S.; Oates, T.W., et al. Antibacterial and remineralizing nanocomposite inhibit root caries biofilms and protect root dentin hardness at the margins. *Journal of dentistry* **2020**, 10.1016/j.jdent.2020.103344, 103344, doi:10.1016/j.jdent.2020.103344.
